# Characteristics of undiagnosed diseases network applicants: implications for referring providers

**DOI:** 10.1186/s12913-018-3458-2

**Published:** 2018-08-22

**Authors:** Nicole M. Walley, Loren D. M. Pena, Stephen R. Hooper, Heidi Cope, Yong-Hui Jiang, Allyn McConkie-Rosell, Camilla Sanders, Kelly Schoch, Rebecca C. Spillmann, Kimberly Strong, Alexa T. McCray, Paul Mazur, Cecilia Esteves, Kimberly LeBlanc, Undiagnosed Diseases Network, David R. Adams, Mercedes E. Alejandro, Patrick Allard, Euan A. Ashley, Mahshid S. Azamian, Carlos A. Bacino, Ashok Balasubramanyam, Hayk Barseghyan, Gabriel F. Batzli, Alan H. Beggs, Hugo J. Bellen, Jonathan A. Bernstein, Anna Bican, David P. Bick, Camille L. Birch, Devon Bonner, Braden E. Boone, Bret L. Bostwick, Lauren C. Briere, Donna M. Brown, Matthew Brush, Elizabeth A. Burke, Lindsay C. Burrage, Shan Chen, Gary D. Clark, Terra R. Coakley, Joy D. Cogan, Cynthia M. Cooper, Heidi Cope, William J. Craigen, Precilla D’Souza, Mariska Davids, Jean M. Davidson, Jyoti G. Dayal, Esteban C. Dell’Angelica, Shweta U. Dhar, Ani Dillon, Katrina M. Dipple, Laurel A. Donnell-Fink, Naghmeh Dorrani, Daniel C. Dorset, Emilie D. Douine, David D. Draper, Annika M. Dries, David J. Eckstein, Lisa T. Emrick, Christine M. Eng, Gregory M. Enns, Ascia Eskin, Cecilia Esteves, Tyra Estwick, Liliana Fernandez, Paul G. Fisher, Brent L. Fogel, Noah D. Friedman, William A. Gahl, Emily Glanton, Rena A. Godfrey, David B. Goldstein, Sarah E. Gould, Jean-Philippe F. Gourdine, Catherine A. Groden, Andrea L. Gropman, Melissa Haendel, Rizwan Hamid, Neil A. Hanchard, Lori H. Handley, Matthew R. Herzog, Ingrid A. Holm, Jason Hom, Ellen M. Howerton, Yong Huang, Howard J. Jacob, Mahim Jain, Yong-hui Jiang, Jean M. Johnston, Angela L. Jones, David M. Koeller, Isaac S. Kohane, Jennefer N. Kohler, Donna M. Krasnewich, Elizabeth L. Krieg, Joel B. Krier, Jennifer E. Kyle, Seema R. Lalani, C. Christopher Lau, Jozef Lazar, Brendan H. Lee, Hane Lee, Shawn E. Levy, Richard A. Lewis, Sharyn A. Lincoln, Allen Lipson, Sandra K. Loo, Joseph Loscalzo, Richard L. Maas, Ellen F. Macnamara, Calum A. MacRae, Valerie V. Maduro, Marta M. Majcherska, May Christine V. Malicdan, Laura A. Mamounas, Teri A. Manolio, Thomas C. Markello, Ronit Marom, Julian A. Martínez-Agosto, Shruti Marwaha, Thomas May, Allyn McConkie-Rosell, Colleen E. McCormack, Alexa T. McCray, Jason D. Merker, Thomas O. Metz, Matthew Might, Paolo M. Moretti, John J. Mulvihill, Jennifer L. Murphy, Donna M. Muzny, Michele E. Nehrebecky, Stan F. Nelson, J. Scott Newberry, John H. Newman, Sarah K. Nicholas, Donna Novacic, Jordan S. Orange, J. Carl Pallais, Christina G. S. Palmer, Jeanette C. Papp, Neil H. Parker, Loren D. M. Pena, John A. Phillips, Jennifer E. Posey, John H. Postlethwait, Lorraine Potocki, Barbara N. Pusey, Chloe M. Reuter, Amy K. Robertson, Lance H. Rodan, Jill A. Rosenfeld, Jacinda B. Sampson, Susan L. Samson, Kelly Schoch, Molly C. Schroeder, Daryl A. Scott, Prashant Sharma, Vandana Shashi, Edwin K. Silverman, Janet S. Sinsheimer, Kevin S. Smith, Ariane G. Soldatos, Rebecca C. Spillmann, Kimberly LeBlanc, Joan M. Stoler, Nicholas Stong, Jennifer A. Sullivan, David A. Sweetser, Cynthia J. Tifft, Camilo Toro, Alyssa A. Tran, Tiina K. Urv, Zaheer M. Valivullah, Eric Vilain, Tiphanie P. Vogel, Daryl M. Waggott, Colleen E. Wahl, Nicole M. Walley, Chris A. Walsh, Michael F. Wangler, Patricia A. Ward, Katrina M. Waters, Bobbie-Jo M. Webb-Robertson, Monte Westerfield, Matthew T. Wheeler, Anastasia L. Wise, Lynne A. Wolfe, Elizabeth A. Worthey, Shinya Yamamoto, Yaping Yang, Guoyun Yu, Diane B. Zastrow, Chunli Zhao, Allison Zheng, Anastasia L. Wise, Vandana Shashi

**Affiliations:** 1 Division of Medical Genetics, Department of Pediatrics, Duke Health, Box 103857, Durham, NC 27710 USA; 2 Department of Allied Health, University of North Carolina at Chapel Hill, Chapel Hill, NC USA; 3 Ethics and Genomics Program, HudsonAlpha Institute for Biotechnology, Huntsville, AL USA; 4 Department of Biomedical Informatics, Harvard Medical School, Boston, MA USA; 5 National Human Genome Research Institute, NIH, Bethesda, MD USA

**Keywords:** Medically unexplained symptoms, Medically unexplained physical symptoms, Undiagnosed diseases, Genomics, Health policy

## Abstract

**Background:**

The majority of undiagnosed diseases manifest with objective findings that warrant further investigation. The Undiagnosed Diseases Network (UDN) receives applications from patients whose symptoms and signs have been intractable to diagnosis; however, many UDN applicants are affected primarily by subjective symptoms such as pain and fatigue. We sought to characterize presenting symptoms, referral sources, and demographic factors of applicants to the UDN to identify factors that may determine application outcome and potentially differentiate between those with undiagnosed diseases (with more objective findings) and those who are less likely to have an undiagnosed disease (more subjective symptoms).

**Methods:**

We used a systematic retrospective review of 151 consecutive Not Accepted and 50 randomly selected Accepted UDN applications. The primary outcome was whether an applicant was Accepted, or Not Accepted, and, if accepted, whether or not a diagnosis was made. Objective and subjective symptoms and information on prior specialty consultations were collected from provider referral letters. Demographic data and decision data on network acceptance were gathered from the UDN online portal.

**Results:**

Fewer objective findings and more subjective symptoms were found in the Not Accepted applications. Not Accepted referrals also were from older individuals, reported a shorter period of illness, and were referred to the UDN by their primary care physicians. All of these differences reached statistical significance in comparison with Accepted applications. The frequency of subspecialty consults for diagnostic purposes prior to UDN application was similar in both groups.

**Conclusions:**

The preponderance of subjective and lack of objective findings in the Not Accepted applications distinguish these from applicants that are accepted for evaluation and diagnostic efforts through the UDN. Not Accepted applicants are referred primarily by their primary care providers after multiple specialist consultations fail to yield answers. Distinguishing between patients with undiagnosed diseases with objective findings and those with primarily subjective findings can delineate patients who would benefit from further diagnostic processes from those who may have functional disorders and need alternative pathways for management of their symptoms.

**Trial registration:**

clinicaltrials.gov NCT02450851, posted May 21st 2015.

**Electronic supplementary material:**

The online version of this article (10.1186/s12913-018-3458-2) contains supplementary material, which is available to authorized users.

## Background

Undiagnosed diseases are defined as constellations of findings that remain refractory to medical diagnostic approaches. Undiagnosed diseases affect approximately 30 million Americans and include (a) rare diseases that are difficult to identify, (b) atypical presentations of known disorders, and (c) yet to be described diseases [1]. Undiagnosed diseases typically manifest with objective findings, which are clinically measurable on physical examination or through medical testing and these provide tangible targets for further diagnostic approaches (e.g. dysmorphic facies, abnormal biochemical profiles, physical exam demonstrating weakness or abnormal gait). Approximately 80% of rare and undiagnosed disorders have a genetic basis [1].

The National Institutes of Health established a multi-site network of clinical sites and core laboratories, the Undiagnosed Diseases Network (UDN), in 2013 [2, 3] (https://undiagnosed.hms.harvard.edu) to facilitate the diagnosis/research of undiagnosed and rare diseases. Network-wide, the UDN receives approximately 70 applications each month from adults and parents of children with unexplained illnesses. Application to the UDN is open to all individuals who complete the application with a referral letter from a healthcare provider. The clinical sites make decisions after a comprehensive review of an applicant’s medical records. The network accepts about half (51.8%) for further evaluation. UDN applicants have a wide range of objective and subjective (non-objective) symptoms; however we noticed that many applicants presented primarily or exclusively with subjective findings.

Subjective findings are non-objective, patient-reported symptoms that are not verifiable by physical exam or medical tests [4–6] (e.g. pain, fatigue, weakness that is not substantiated by physical exam). Patients with subjective findings may fall under the diagnostic terms of functional disorder, central sensitivity syndrome, subjective health complaints or even carry the DSM5 diagnosis of somatic symptom disorder. It is difficult to estimate the number of patients affected by these disorders, although it represents a common conundrum in adult primary care medicine [7–9]. Many patients with primarily/exclusively subjective findings may consider themselves to have undiagnosed diseases and while a small number may have underlying organic disease, the majority are not believed to do so [6, 10].

Both patients with objective and subjective findings accrue substantial healthcare costs and seek opinions from multiple specialists, often undergoing extensive/invasive diagnostic testing in their search for a diagnosis [11–14]. Both groups also express chaos in their lives, frustration with negative laboratory tests/procedures, and seek validation of their symptoms [15]. This results in significant personal psychosocial and financial distress and a societal economic impact in both entities [16–19]. However, the likely underlying causes for each group are different: Subjective findings have a number of predisposing risk factors and environmental triggers [20, 21] and genetic factors are expected to play at most a modest role [22]. Conversely, approximately 80% of undiagnosed disorders with clear objective findings have a genetic etiology [1, 17, 23]. Thus, the diagnostic and management approaches for each group differ, with strategies focused on symptom management for the subjective group and further diagnostic pathways, including genomic sequencing for undiagnosed diseases with objective findings.

Utilizing the UDN applicant cohort, we characterize symptoms, referral patterns, and demographic factors among applicants that are and are not accepted for evaluation in the UDN. We sought to provide information to healthcare providers and patients regarding applicants that are suitable for further diagnostic avenues such as the UDN, but more importantly we hope to contribute to a discussion on the need for diagnostic/management/research pathways for applicants who suffer primarily/exclusively from subjective findings.

## Methods

We retrospectively reviewed 201 UDN applications: 151 consecutive applications that were not accepted (Not Accepted Applicants) and 50 randomly selected applications that were accepted (Accepted Applicants) across the seven UDN clinical sites (Additional file 1: Table S1). All applicants provided electronic informed consent as approved by the National Human Genome Research Institute Institutional Review Board under research protocol 15-HG-0130.

Data were provided by the UDN Coordinating Center from the online portal wherein network-wide information is stored. Applications were included if the application decision was issued between 3/1/2016 and 1/3/2017. We excluded applications that were still under review by the clinical sites and those that lacked a referral letter from a healthcare provider. For all applications, we recorded application outcome, applicant demographics, and application metrics (e.g. referring healthcare provider data). From study referral letters we recorded the number and type of specialty consultations (if any) that were specifically mentioned and medical information described by healthcare providers. For Not Accepted Applicants only, we recorded reasons for non-acceptance and recommendations from the clinical site to the applicant/referring healthcare provider (Additional file 1: Table S2).

On an initial review of 30 Not Accepted Applications, we identified subjective symptoms that were commonly mentioned in referral letters (Additional file 1: Table S3). These were modified through consensus discussions by three authors (NMW, LDMP, VS) and then extracted from all the letters. Objective findings varied greatly and were tallied according to their presence in pertinent organ systems (Additional file 1: Table S4). Findings were considered to be objective if the healthcare provider mentioned an abnormal test or examination finding by name. For example, muscle weakness counted as an objective finding only if the provider documented that the weakness was present on physical exam. If specific diagnoses, (e.g. fibromyalgia) were mentioned, these were recorded separately.

Referring healthcare providers included physicians and advance practice providers (e.g. physician assistants and nurse practitioners) who wrote the UDN referral letter. Physicians were classified as primary care physicians or specific specialty when they identified themselves as such, provided letterhead that included this information, or were found via web search to practice family medicine or general internal medicine. Advance practice providers were included in the category “Other” irrespective of their specialty.

Statistical analyses were performed using SPSS 24.0. We used descriptive analyses, student’s T-test, Fisher’s exact test (FET), ANOVA, and chi-square analyses to analyze factors that affected application outcome. Post-hoc analyses were performed when indicated to infer the significance and direction of individual variables. Binary logistic regression identified associations between the dependent variable of application outcome and key demographic and medical factors.

## Results

### Demographics

All UDN clinical sites were represented for the Not Accepted and Accepted applications (Additional file 1: Table S1). There were no significant differences between the Accepted and Not Accepted applicants for gender, race or ethnicity. The Accepted cohort was significantly younger at the time of application and had earlier onset of illness. The duration of illness in both groups was similar but the proportion of an applicant’s life being ill was significantly higher for the Accepted applicants. The Not Accepted applications required a significantly longer amount of time for application and medical record review (Table 1).

**Table 1:** Demographic data of Not Accepted applicants compared to Accepted applicants

Feature	Not Accepted (*n* = 151)	Accepted (*n* = 50)	Statistics
Age at Application (years, mean ± SD)	39.02 ± 19.11	21.03 ± 18.83	*t* = 5.78** Cohen’s *d* = 0.94
Age at Symptom Onset (years, mean ± SD)	30.31 ± 18.75	11.11 ± 18.0	*t* = 6.3** Cohen’s *d* = 1.0
Duration of Illness (years, mean ± SD)	8.71 ± 9.91	9.92 ± 10.06	*t* = −0.75 Cohen’s *d* = − 0.12
Proportion of Lifetime Being Ill (percentage)	28.31 ± 29.79	66.69 ± 37.08	*t* = −6.6** Cohen’s *d* = −1.14
Length of Application Review (months, mean ± SD)	3.4 ± 1.73	2.78 ± 1.76	*t* = 2.17* Cohen’s *d* = 0.35
Gender			
Female	79 (52%)	26 (52%)	FET = 1.0
Male	72 (48%)	24 (48%)	
Race			
White	128 (84.7%)	42 (84%)	χ^2^ = 0.10
African-American	7 (4.6%)	2 (4%)	
Other	16 (10.7%)	6 (12%)	
Ethnicity			
Hispanic	11 (7.3%)	5 (10%)	χ^2^ = 0.38
Non-Hispanic	124 (82.1%)	40 (80%)	
Not reported	16 (10.6%)	5 (10%)	

*SD* Standard deviation, *FET* Fisher’s exact test

All t-tests were two-tailed *p < 0.05 **p < 0.001

### Healthcare provider referral patterns

**Fig. 1:**
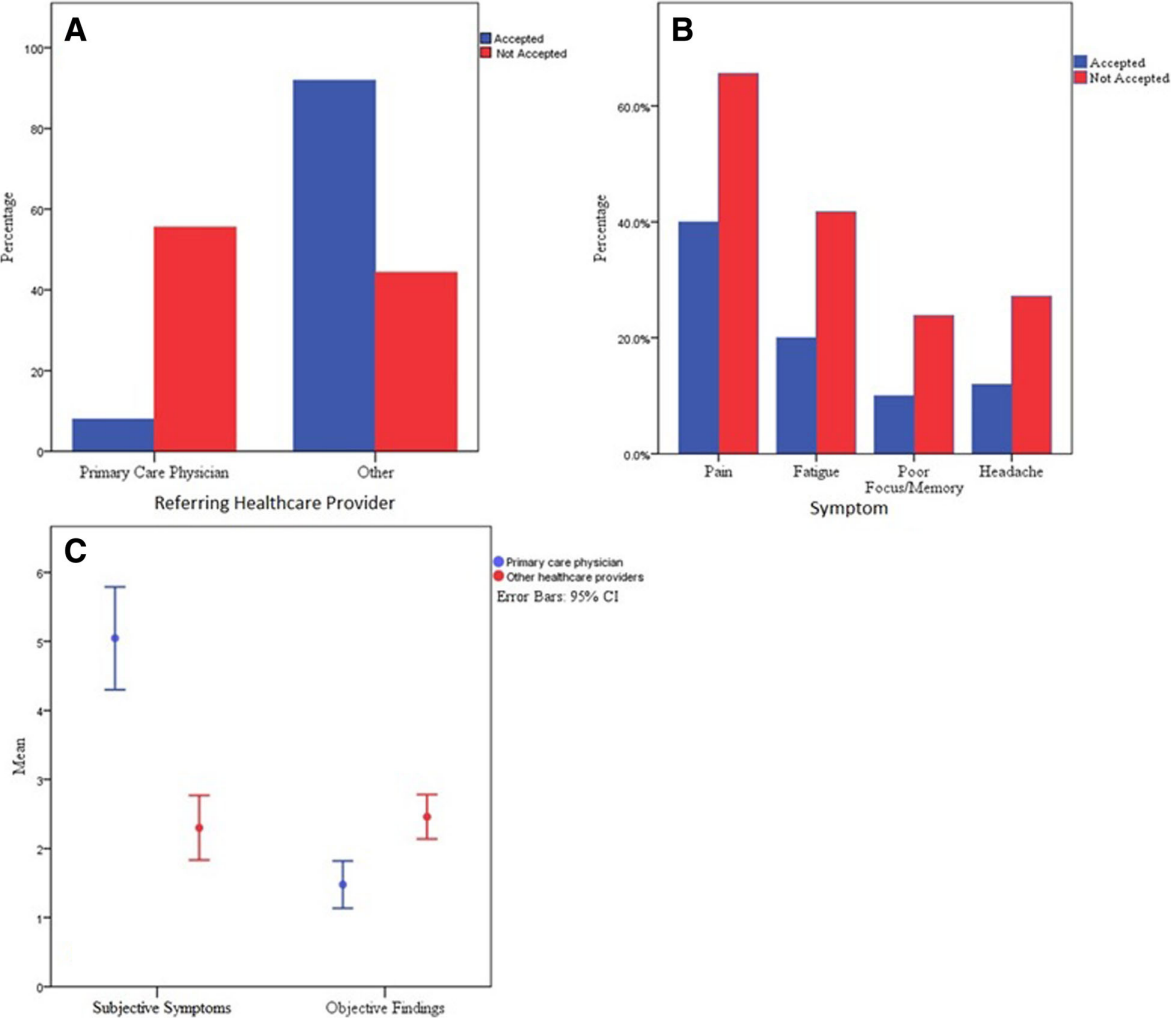
Referral sources and common symptoms in UDN applications . 1A: Not Accepted individuals were significantly more likely to be referred by their primary care physicians than healthcare providers in all other disciplines combined (FET, *p* < 0.001). 1B: The four most often reported symptoms in the Not Accepted group are reported significantly less frequently in the Accepted group (χ2 = 15.43, *p* < 0.01). 1C: Referral letters authored by primary care physicians mentioned significantly more subjective symptoms (*t* = 6.2, *p* < 0.001) and fewer objective findings (*t* = − 4.15, *p* < 0.001) than letters authored by other providers regardless of application outcome

The most common source of referrals to the UDN were primary care physicians (PCP) (88/201, 43.7%). PCP referrals accounted for significantly more applications that were ultimately not accepted [55.6% of Not Accepted applicants, 8.0% of Accepted Applicants, FET *p* < 0.001 (Table 2, Fig. 1a)]. Accepted Applicants were referred most often by geneticists or neurologists (66%, Table 2). We found no significant group differences in the number of specialist consultations noted by study referral letters (Accepted: 2.62 ± 2.66, Not Accepted: 3.12 ± 2.67, *t* = − 1.14, *p* = 0.252, Cohen’s *d* effect size = 0.18).

**Table 2:** Applicant referral sources for all applicants and Reasons for Non-Acceptance for the Not Accepted applications

Healthcare Provider	Not Accepted	Accepted	Statistics
Primary Care Physicians compared to other Specialty Healthcare Providers			
Primary Care Physician	84 (55.6%)^a^	4 (8.00%)^a^	χ^2^ = 55.48, *p* < 0.001
Specialist: Neurologist	18 (11.9%)	14 (28.0%)^a^	
Specialist: Geneticist	8 (5.3%)^a^	19 (38.0%)^a^	
Other (e.g. Allergy Immunologist, Rheumatologist)	41 (27.2%)	13 (26%)	
Primary Care Physicians compared to all Other Healthcare Providers			
Primary Care Physicians	84 (55.6%)	4 (8%)	FET *p* < 0.001
Others	67 (44.3%)	46 (92%)	

^a^Significant adjusted residuals exceeding +/− 2 on post-hoc testing

*FET* Fisher’s exact test

### Subjective symptoms and objective findings

The mean number of subjective symptoms was higher in the Not Accepted applicants (4.1 ± 3.37) relative to the Accepted (1.7 ± 2.10, *t* = 4.7, *p* < 0.001, Cohen’s *d* effect size = 0.85). This remains true for subjective symptoms when Accepted applicants are stratified according to whether they did (*N* = 16) or did not (*N* = 34) receive a diagnosis in the UDN (Not Accepted: mean 4.1 ± 3.37, Accepted-Not Diagnosed mean 2.06 ± 2.33, Accepted-Diagnosed mean 0.94 ± 1.39 (ANOVA F = 11.9, *p* < 0.001)). The four most common subjective symptoms (pain, fatigue, headache and memory problems (Additional file 1: Table S3)) were present significantly less frequently in applicants in the Accepted cohort (χ^2^ = 15.43, *p* < 0.01) (Fig. 1b). The majority (114/151, 75.5%) of the Not Accepted applicants had at least one objective clinical finding but the mean number of objective findings in the Not Accepted group (1.67 ± 1.6) was significantly lower than in the Accepted group (3.12 ± 1.53, *t* = − 5.4, *p* < 0.001, Cohen’s *d* = − 0.92). This finding remained true when Accepted applicants are again stratified by diagnosis (Not Accepted: mean 1.67 ± 1.64, Accepted-Not Diagnosed mean 3.21 ± 1.72, Accepted-Diagnosed mean 2.94 ± 1.24 (ANOVA F = 15.0, *p* < 0.001)). In the entire cohort of 201 applicants, there were significantly more subjective symptoms in applicants referred by a primary care physician (*n* = 88, 5.05 ± 3.50) than other healthcare providers (*n* = 113, 2.20 ± 2.50, *t* = 6.2, *p* < 0.001, Cohen’s *d* = 0.93) (Fig. 1c) and significantly fewer objective findings in those referred by a primary care physician (1.48 ± 1.61) than other healthcare providers (2.46 ± 1.71, *t* = − 4.15, *p* < 0.001, Cohen’s *d* = 0.6) (Fig. 1c).

### Prior diagnoses

Specific diagnoses were mentioned in some Not Accepted referral letters and the top four: Fibromyalgia, Chronic Fatigue Syndrome, Mitochondrial disorder and Lyme disease were collectively reported in 28/151 (18.5%) Not Accepted applications, compared to 6/50 Accepted applications (12%) (FET *p* > 0.05). However, the individual diagnosis of fibromyalgia was reported significantly more often in the Not Accepted applications (16/151, 10.6%) relative to the Accepted (1/50, 2%, FET *p* = < 0.05). For the other three diagnoses, there were no significant group differences (FET *p*= > 0.05), with a frequency of 0–9% in both groups (Additional file 1: Figure S1).

#### Reasons for non-acceptance

Not Accepted applicants were given reasons for this decision, the most common being that the UDN was unlikely to make a diagnosis, likely reflective of the lack of objective findings in referral letters (77/151, 51.0%). Specific recommendations were provided to 33.8% (51/151) of the Not Accepted applicants, including recommendations to pursue specific diagnostic tests and seek subspecialty care.

#### Categorization of illness

Neurology was the most common system category selected by both Accepted (24/50, 48%) and Not Accepted applicants (44/151, 29.1%, FET *p* < 0.05), followed by Allergy-Immunology (8% in Accepted and 10.5% in Not Accepted) and Musculoskeletal (12% in Accepted and 7.9% in Not Accepted). No applicant in either group chose Psychiatry as their disease category; objective psychiatric findings were documented in three Not Accepted healthcare provider letters (Additional file 1: Table S4).

#### Outcome associations

On binary logistic regression with the referring healthcare provider specialty, age at application and age at symptom onset as independent variables and application outcome as the dependent variable, a primary care referral was significantly associated with non-acceptance. Age at application and age at symptom onset were not significantly associated with acceptance status. Similarly, binary logistic regression with the presenting findings showed that higher number of objective symptoms and fewer subjective symptoms were significantly associated with being accepted (Table 3).

**Table 3:** Logistic regression demonstrating relation between key demographic and medical variables and Acceptance or Non-Acceptance into the UDN

Criterion	Odds ratio	95% Confidence Interval
Demographics		
Age at UDN Application	1.01	0.98–1.05
Age at onset of symptoms	1.03	0.99–1.07
Referring healthcare provider (Primary care or Other)	8.86***	2.84–26.5
Medical Findings		
Subjective Symptoms	1.40***	1.19–1.66
Objective Findings	0.62***	0.5–0.76

*p < 0.05, **p < 0.01, ***p < 0.001

## Discussion

Our findings indicate that many applicants that are not accepted to the UDN suffer primarily from subjective symptoms. Further, Not Accepted Applicants were more often adults who had experienced illness for a shorter proportion of their lives and had more non-neurological presentations. Although objective and subjective symptoms were seen in both groups, higher numbers of subjective symptoms were reported for Not Accepted Applicants relative to Accepted subjects. This is true regardless of whether an Accepted applicant was diagnosed by the UDN or not, indicating that a functional disorder was less likely in these remaining still-undiagnosed Accepted applicants, distinguishing them from the Not Accepted applicants. When objective findings were noted in the Not Accepted applicants, these were often unrelated to the reasons for applying to the UDN (e.g. iron deficiency anemia, abnormal thyroid profile) or were non-specific (e.g. positive anti-nuclear antibody). Taken together, we infer that Not Accepted subjects suffer primarily from subjective symptoms that are unlikely to be caused by or related to any objective findings. As such, these applicants are not candidates for further diagnostic efforts such as those offered by the UDN. Interestingly, the Not Accepted Applications took longer to review, perhaps due to a higher volume of medical records that needed to be reviewed to determine if there were features (such as objective findings beyond subjective symptoms) that would warrant acceptance, although we specifically did not ascertain the reasons for review time.

We also found that primary care physicians referred the largest proportion of applicants to the UDN, but that their referrals had fewer objective findings and were less likely to be accepted. We did not identify a clear reason for this finding, but given that there is no difference in the number of specialty consultations between Not Accepted Applicants and Accepted Applicants, a lack of access to specialists is unlikely. Instead, we presume this indicates that patients with non-objective symptoms continue to seek diagnostic efforts and additional referrals through their PCPs after consultations with specialists do not provide a diagnosis [24].

In the context of the UDN, and perhaps more importantly in the setting of clinical practice, it is important to make a distinction between undiagnosed patients with and without objective findings. Undiagnosed patients with clear objective findings are expected to have a genetic basis in most instances; therefore these diseases can reasonably be expected to be solved (eventually) with additional tests, procedures, and genomic investigations. The UDN is one of several avenues available to pursue a unifying diagnosis. For patients with primarily/exclusively subjective symptoms, additional clinical or genomic investigations are likely to provide a concrete diagnosis in only a small minority (< 5%) of cases [10, 25]. There is evidence that risk factors may play a part, such as stress, underlying mood disorders and viral infections, and there are several diagnostic terms, including “functional disorder,” “central sensitivity syndrome,” or “somatic symptom disorder” that may fit these individuals [20, 21]. Whether these diagnoses have been discussed with these UDN applicants is not ascertainable from the data available, but it is certain that these applicants attribute their symptoms to a physical illness that requires additional diagnostic efforts.

Despite the dichotomy between undiagnosed patients with and without objective symptoms, the societal and personal impact is remarkably similar. Both groups incur high medical costs and the affected individuals are often significantly disabled. In our recent analysis of UDN narratives, all applicants expressed similar levels of chaos in their lives and frustration with negative laboratory tests/procedures and many stated that the UDN represented their last hopes for a diagnosis [15].

Although the UDN provides recommendations for applicants and their physicians whenever appropriate, in many cases the onus of continuing medical investigation and treatment falls to primary care physicians, who themselves have limited resources, especially limited time, and few research avenues available to them in their efforts to diagnose/treat patients with subjective findings. There are some research studies that may be an option (https://mecfs.ctss.nih.gov; https://painconsortium.nih.gov), but there are only a few specific clinical programs (http://www.chop.edu/centers-programs/center-amplified-musculoskeletal-pain-syndrome; http://www.amazingkids.org/Medical-Services/pain-rehabilitation) to address non-objective symptoms, and they are treat primarily children. The diagnostic terms “functional disorder,” “central sensitivity syndrome,” or “somatic symptom disorder” were almost never identified in referral letters, indicating that PCPs may not be discussing these. Clearly more avenues for management of subjective symptoms are needed beyond the care that PCPs may be able to provide these patients.

This study has some limitations that future research efforts may address. We were unable to ascertain a comprehensive list of subjective/objective findings directly from medical records review. We may have under-ascertained primary care provider referrals because all mid-level providers were classified as “other” regardless of specialty. We were unable to speak with referring providers directly, and future studies that consider these discussions may provide additional insights into a physician’s motivations for referral. We were not able to follow applicants after a decision was made and so do not know if any of the Not Accepted applicants received a diagnosis. We also did not have socioeconomic status data available and these may have influenced the types of applications received.

## Conclusions

In conclusion, making a distinction between undiagnosed diseases with and without objective findings enable the affected individuals and their providers, who very often are PCPs, to identify the correct path forward for further management. Confusing subjective symptoms for undiagnosed diseases can lead to significant loss of time and resources to identify diagnoses, when further management of the symptoms would be more optimal. Given the prevalence of subjective findings in the general population additional research is warranted in this area to provide clinically appropriate diagnostic guidelines, establish evidence-based treatment options and facilitate honest and effective communication about these illnesses with the patients.

## Additional file

Additional file 1: **Figure S1.** Referral sources and common symptoms in UDN applications. 1A: Not Accepted individuals were significantly more likely to be referred by their primary care physicians than healthcare providers in all other disciplines combined (FET, *p* < 0.001). 1B: The four most often reported symptoms in the Not Accepted group are reported significantly less frequently in the Accepted group (χ2 = 15.43, *p* < 0.01). 1C: Referral letters authored by primary care physicians mentioned significantly more subjective symptoms (*t* = 6.2, *p* < 0.001) and fewer objective findings (*t* = − 4.15, *p* < 0.001) than letters authored by other providers regardless of application outcome. These materials contain 4 additional data tables and one supplementary figure. (DOCX 63 kb)

## Abbreviations

PCP: Primary Care Physician; FET, Fisher’s Exact Test
UDN: Undiagnosed Diseases Network
