# Supplementary material for: Characteristics of undiagnosed diseases network applicants: implications for referring providers
Source: BMC Health Serv Res. 2018 Aug 22;18:652. doi: 10.1186/s12913-018-3458-2 (PMC6106923; doi:10.1186/s12913-018-3458-2)
Supplement: Supplementary file 1 — Figure S1. Referral sources and common symptoms in UDN applications. 1A: Not Accepted individuals were significantly more likely to be referred by their primary care physicians than healthcare providers in all other disciplines combined (FET, p < 0.001). 1B: The four most often reported symptoms in the Not Accepted group are reported significantly less frequently in the Accepted group (χ2 = 15.43, p < 0.01). 1C: Referral letters authored by primary care physicians mentioned significantly more subjective symptoms (t = 6.2, p < 0.001) and fewer objective findings (t = − 4.15, p < 0.001) than letters authored by other providers regardless of application outcome. These materials contain 4 additional data tables and one supplementary figure. (DOCX 63 kb) [file 12913_2018_3458_MOESM1_ESM.docx]

Supplemental Materials

Table S1: UDN clinical sites from which applications were reviewed

| **Assigned UDN site** | **Not Accepted** | **Accepted** | **All** |
| --- | --- | --- | --- |
| Baylor | 15 | 7 | 22 |
| Duke | 31 | 7 | 38 |
| Harvard | 18 | 7 | 25 |
| NIH UDP | 27 | 8 | 35 |
| Stanford | 7 | 7 | 14 |
| UCLA | 21 | 7 | 28 |
| Vanderbilt | 32 | 7 | 39 |
| Total | 151 | 50 | 201 |

Table S2: Details of data collected on applicants Not Accepted (n=151) and Accepted (n=50)

| Data Points | |
| --- | --- |
| Demographic Information | Race |
|  | Ethnicity |
|  | Gender |
|  | Age at Application |
|  | Age at Symptom Onset |
| Application Metrics | Assigned Clinical Site |
|  | Length of Application Review |
|  | Application Outcome (Decision Letter from UDN Clinical Site) |
|  | Recommendations Provided |
|  | Primary Symptom Category (Applicant Identified) |
|  | Referring Healthcare Provider Specialty |
|  | Presence/Absence of Objective Findings in Healthcare Provider Recommendation Letter |
| Subjective Symptoms in Healthcare Provider Referral Letter as Reason(s) for Referral | 33 symptoms commonly reported, see Supplementary Table S3 |
| Specific Diagnoses in Healthcare Provider Referral Letter | Fibromyalgia |
|  | Chronic Fatigue Syndrome |
|  | Mitochondrial/Presumed Mitochondrial Disorder |
|  | Lyme Disease |
|  | Other Specific/Correct Diagnosis |
| Objective Findings in Healthcare Provider Referral Letter | Present or absent in 22 different organ systems, see Supplementary Table S4 |
| Prior Specialty Consultations note in Healthcare Provider Referral Letter | Specialty consultations noted specifically by the referring provider. |

Table S3: Subjective symptoms commonly reported in healthcare provider referral letters

| **Symptom Noted in Referral Letter** | **Count – Not Accepted** | **Count – Accepted** |
| --- | --- | --- |
| Pain | 99 (65.56%) | 20 (40.00%) |
| Fatigue | 63 (41.72%) | 10 (20.00%) |
| Headache | 41 (27.15%) | 6 (12.00%) |
| Difficulty Thinking/Focusing, Memory Problems | 36 (23.84%) | 5 (10.00%) |
| Nausea | 29 (19.21%) | 4 (8.00%) |
| Unable/Unwilling to Work or Maintain Employment / Out of Work | 29 (19.21%) | 1 (2.00%) |
| Muscle Weakness | 25 (16.56%) | 12 (24.00%) |
| Disability / Disabled / Disabling Condition (without implication of patient receiving benefits) | 24 (15.89%) | 0 (0.00%) |
| Depression | 23 (15.23%) | 1 (2.00%) |
| Anxiety | 22 (14.57%) | 2 (4.00%) |
| Tingling | 21 (13.91%) | 4 (8.00%) |
| Dizziness | 20 (13.25%) | 1 (2.00%) |
| Difficulty Breathing | 19 (12.58%) | 2 (4.00%) |
| Insomnia/Difficulty Sleeping | 18 (11.92%) | 0 (0.00%) |
| Weight Loss | 15 (9.93%) | 3 (6.00%) |
| Constipation | 12 (7.95%) | 3 (6.00%) |
| Fainting/Passing Out/Altered Consciousness | 12 (7.95%) | 2 (4.00%) |
| Speech abnormality or difficulty | 11 (7.28%) | 1 (2.00%) |
| Temperature Intolerance, Heat/Cold Sensitivity | 11 (7.28%) | 0 (0.00%) |
| Light Sensitivity/Photophobia | 10 (6.62%) | 2 (4.00%) |
| Burning | 10 (6.62%) | 0 (0.00%) |
| Food Allergies | 10 (6.62%) | 0 (0.00%) |
| Blurry/Double Vision | 9 (5.96%) | 0 (0.00%) |
| Throat Constriction/Difficult Swallowing | 8 (5.30%) | 0 (0.00%) |
| Ringing in Ears | 7 (4.64%) | 0 (0.00%) |
| Loss of Appetite | 6 (3.97%) | 2 (4.00%) |
| Weight Gain | 6 (3.97%) | 1 (2.00%) |
| Noise Sensitivity/Phonophobia | 6 (3.97%) | 1 (2.00%) |
| Bloated | 5 (3.31%) | 0 (0.00%) |
| Hair Loss | 4 (2.65%) | 1 (2.00%) |
| On Disability (implies receiving disability benefits) | 4 (2.65%) | 1 (2.00%) |
| Tiredness/Somnolence/Sleepiness | 3 (1.99%) | 0 (0.00%) |
| Increased Appetite | 1 (0.66%) | 0 (0.00%) |

Table S4: Organ systems under which objective findings from healthcare provider referral letter were categorized

|  | Not Accepted Applicants | | Accepted Applicants | |
| --- | --- | --- | --- | --- |
|  | Patient-Reported Symptom Category | Objective Findings Noted in Referral Letter | Patient-Reported Symptom Category | Objective Findings Noted in Referral Letter |
| Allergies and Disorders of The Immune System | 16 (10.60%) | 19 (12.58%) | 4 (8.00%) | 10 (20.00%) |
| Cardiology and vascular conditions | 1 (0.66%) | 28 (18.54%) | 2 (4.00%) | 11 (22.00%) |
| Dentistry and craniofacial | 1 (0.66%) | 2 (1.32%) | 0 (0.00%) | 4 (8.00%) |
| Dermatology | 2 (1.32%) | 7 (4.63%) | 1 (2.00%) | 5 (10.00%) |
| Endocrinology | 4 (2.65%) | 21 (13.90%) | 1 (2.00%) | 11 (22.00%) |
| Gastroenterology | 10 (6.62%) | 30 (19.87%) | 1 (2.00%) | 13 (26.00%) |
| Genetics | 0 (0.00%) | 7 (4.63%) | 0 (0.00%) | 17 (34.00%) |
| Gynecology and reproductive medicine | 1 (0.66%) | 5 (3.31%) | 0 (0.00%) | 0 (0.00%) |
| Hematology | 3 (1.99%) | 10 (6.62%) | 0 (0.00%) | 5 (10.00%) |
| Infectious Diseases | 2 (1.32%) | 8 (5.30%) | 0 (0.00%) | 2 (4.00%) |
| Musculoskeletal and orthopedics | 12 (7.95%) | 26 (17.22%) | 6 (12.00%) | 14 (28.00%) |
| Nephrology | 0 (0.00%) | 4 (2.65%) | 0 (0.00%) | 6 (12.00%) |
| N/A | 19 (12.58%) | 0 (0.00%) | 1 (2.00%) | 0 (0.00%) |
| Neurology | 44 (29.14%) | 43 (28.5%) | 24 (48.00%) | 39 (78.00%) |
| Oncology | 1 (0.66%) | 4 (2.65%) | 0 (0.00%) | 1 (2.00%) |
| Ophthalmology | 1 (0.66%) | 7 (4.63%) | 0 (0.00%) | 10 (20.00%) |
| Other | 16 (10.60%) | 0 (0.00%) | 5 (10.00%) | 4 (8.00%) |
| Psychiatry | 0 (0.00%) | 3 (1.99%) | 0 (0.00%) | 1 (2.00%) |
| Pulmonology | 6 (3.97%) | 11 (7.28%) | 0 (0.00%) | 3 (6.00%) |
| Rheumatology | 10 (6.62%) | 13 (8.61%) | 5 (10.00%) | 7 (14.00%) |
| Toxicology and Environmental Medicine | 1 (0.66%) | 0 (0.00%) | 0 (0.00%) | 0 (0.00%) |
| Urology | 1 (0.66%) | 4 (2.65%) | 0 (0.00%) | 3 (6.00%) |

Figure S1: Frequency of the top four diagnoses mentioned in the healthcare provider referral letters.


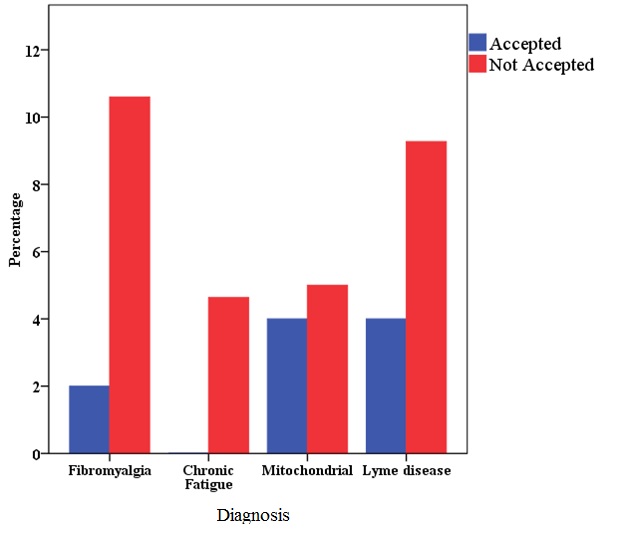


The diagnosis of fibromyalgia was reported significantly more often in the Not Accepted applications (16/151, 10.6%) relative to the Accepted (1/50, 2%, FET p=<0.05). No other diagnoses were reported with significantly different frequencies between the two groups.
